# Active transforming growth factor-β is associated with phenotypic changes in granulomas after drug treatment in pulmonary tuberculosis

**DOI:** 10.1186/s13069-016-0043-3

**Published:** 2016-04-27

**Authors:** Robert M. DiFazio, Joshua T. Mattila, Edwin C. Klein, Lauren R. Cirrincione, Mondraya Howard, Eileen A. Wong, JoAnne L. Flynn

**Affiliations:** Department of Microbiology and Molecular Genetics, University of Pittsburgh School of Medicine, Pittsburgh, PA 15261 USA; Department of Infectious Diseases and Microbiology, Pitt Public Health, University of Pittsburgh, Pittsburgh, PA 15261 USA; Division of Laboratory Animal Resources, University of Pittsburgh, Pittsburgh, PA 15261 USA; University of Pittsburgh School of Pharmacy, Pittsburgh, PA 15261 USA

**Keywords:** Tuberculosis, Drug treatment, Transforming growth factor-β, Collagen I

## Abstract

**Background:**

Tuberculosis (TB) chemotherapy clears bacterial burden in the lungs of patients and allows the tuberculous lesions to heal through a fibrotic process. The healing process leaves pulmonary scar tissue that can impair lung function. The goal of this study was to identify fibrotic mediators as a stepping-stone to begin exploring mechanisms of tissue repair in TB.

**Methods:**

Hematoxylin and eosin staining and Masson’s trichrome stain were utilized to determine levels of collagenization in tuberculous granulomas from non-human primates. Immunohistochemistry was then employed to further interrogate these granulomas for markers associated with fibrogenesis, including transforming growth factor-β (TGFβ), α-smooth muscle actin (αSMA), phosphorylated SMAD-2/3, and CD163. These markers were compared across states of drug treatment using one-way ANOVA, and Pearson’s test was used to determine the association of these markers with one another.

**Results:**

TGFβ and αSMA were present in granulomas from primates with active TB disease. These molecules were reduced in abundance after TB chemotherapy. Phosphorylated SMAD-2/3, a signaling intermediate of TGFβ, was observed in greater amounts after 1 month of drug treatment than in active disease, suggesting that this particular pathway is blocked in active disease. Collagen production during tissue repair is strongly associated with TGFβ in this model, but not with CD163+ macrophages.

**Conclusions:**

Tissue repair and fibrosis in TB that occurs during drug treatment is associated with active TGFβ that is produced during active disease. Further work will identify mechanisms of fibrosis and work towards mitigating lung impairment with treatments that target those mechanisms.

**Electronic supplementary material:**

The online version of this article (doi:10.1186/s13069-016-0043-3) contains supplementary material, which is available to authorized users.

## Background

With eight million new cases and 1.5 million deaths annually worldwide, tuberculosis (TB) is one of the humanity’s greatest health threats [1]. Granulomas, the pathologic hallmark of TB, are well-circumscribed organized collections of host immune cells that form in response to the inhalation of aerosols containing *Mycobacterium tuberculosis* (Mtb)—the causative agent of TB. Although granulomas can function to kill or contain Mtb, they can also serve as a niche for growth and persistence of the organism [2–4]. Granulomas often feature a necrotic center and are thus dubbed as necrotizing or caseous, while granulomas lacking this necrosis are said to be non-necrotizing. Granulomas feature epithelioid macrophages, elongated cells with larger nuclei, surrounded by other macrophages and lymphocytes [5]. Bacteria can be found in epithelioid macrophages and in caseum [5]. Uncontrolled replication leads to dissemination of the bacteria and formation of new granulomas. However, some granulomas can restrain bacterial dissemination or even develop locally sterilizing immunity. As a result, these granulomas are often fibrotic and can contain a calcified core (referred to as fibrocalcific lesions) [6]. A mixture of necrotizing and collagenous lesions is typical of the secondary pulmonary tuberculosis and is referred to as fibrocaseous disease [6, 7]. Although the host factors that lead to control or dissemination of a single granuloma are unclear, we have demonstrated that various types of granulomas and outcomes exist within a single non-human primate, similar to humans [6, 8].

Tissue fibrosis can result from a wound healing response that includes fibroblast activation and recruitment, production of extracellular matrix materials, and distortion of the normal tissue architecture. The most common extracellular matrix component is collagen I, which is the most fibrous form of collagen and represents about 84 % of the collagen produced by fibroblasts [9]. Fibrosis can be caused by a local inflammatory response, and fibrosis-related pathogenesis is associated with dysfunction of many organs, including lungs, liver, and kidneys [10–12]. Transforming growth factor-β (TGFβ) is the main cytokine implicated in fibrogenesis, although other cytokines are implicated, including TNF, IL-6, IL-10, IL-13, and IL-17 [13–18]. TGFβ is produced in a latent form (L-TGFβ) and can be activated through the plasmin protease pathway, CD36 and thrombospondin (TSP), reactive oxygen and nitrogen species, hypoxia, low pH, and matrix metalloproteases [19]. Active TGFβ utilizes type 1 and 2 TGFβ receptors, signaling through a variety of intermediaries, including phosphorylated SMAD-2/3 [20]. Through these intermediaries, TGFβ stimulates differentiation of fibroblasts into myofibroblasts that then produce alpha-smooth muscle actin (αSMA), a key indicator of and contributor to fibrotic pathogenesis [21]. TGFβ has been observed and measured in pulmonary fibrosis and, in the lung, is produced by alveolar macrophages, fibrocytes, and lung epithelial cells [22–25]. Alveolar macrophages from humans with pulmonary fibrosis display an alternatively activated (M2) phenotype, and induction and maintenance of M2 macrophages is critical to pathology in pulmonary fibrosis [26]. M2 macrophages are also major producers and activators of TGFβ [24].

Although several types of pulmonary fibrosis have been characterized and studied, fibrosis in tuberculosis is not well-understood. Significant pulmonary impairment was observed in 59 % of patients with TB disease [27], half of whom had less than 50 % of their original forced vital capacity [27]. This loss of pulmonary function resulted in 177 subjects losing 1189 disability adjusted-life years [28]. Lung function does not improve over the course of chemotherapy [29], and this chronic impairment increases incrementally with the number of TB episodes experienced in a progressive manner [29]. The main course of treatment for post-tuberculosis lung damage is pulmonary rehabilitation, which has mixed results [30], highlighting the need for more targeted therapies to resolve TB-induced fibrosis and scarring. Since macrophages produce TGFβ in pulmonary fibrosis, and are a major cellular component of granulomas [24], macrophages may be important contributors to fibrosis in TB lesions [31]. The environment of the granuloma may contain almost all of the conditions that activate TGFβ, including hypoxia [32], nitrogen radicals [5], and metalloproteases [33], so it is likely that the disease process activates TGFβ locally at the site of infection. Cutaneous TB lesions in humans have been noted as centers of fibrosis, with lesions containing active TGFβ [34]. Patients with TB have peripheral blood monocytes and alveolar macrophages that produce and active more TGFβ than cells from healthy controls [35, 36]. TGFβ has also been observed directly in granulomas from human TB patients by immunohistochemistry [37].

Drug treatment for *M. tuberculosis* infection [38, 39] is a lengthy process that slowly clears bacterial burden in the lung and induces tissue repair in TB-affected lung. The factors that promote fibrotic resolution of tuberculous granulomas are poorly understood. This has been a challenging topic to address because of difficulties associated with studying human TB and a lack of appropriate mouse models demonstrating the granuloma structures seen in humans. Our laboratory previously published that drug-treated macaques with TB had fibrotic granulomas, and the fibrotic granulomas were most often sterile [40], representing a successful outcome of drug treatment. Since macaques recapitulate the spectrum of granuloma types and infection outcomes seen in humans, they represent a useful system for studying the process of drug-associated fibrosis. Understanding the fibrotic processes that occur in TB may provide insights into treatments to safely resolve residual lung fibrosis during or after drug therapy. The objective of this study was to determine how the cell types and molecules associated with pulmonary fibrosis differ between granulomas associated with active TB and fibrotic changes after chemotherapy. This study will open up further exploration of the fibrogenic mechanisms, with the aim of developing treatments to minimalize or reverse scarring after drug treatment.

## Results

### Macrophages experience spindloid transformation in tuberculous granulomas

**Fig. 1:**
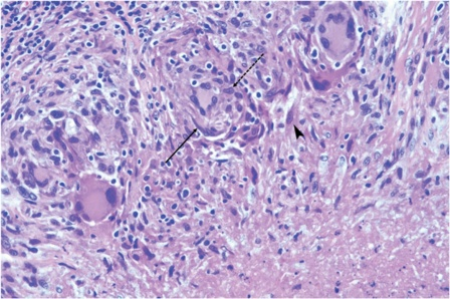
Macrophages experience spindloid transformation in tuberculous granulomas. H&E stained granuloma that demonstrates spindloid transformation of histiocytes. This figure provides microhistological examples of: epithelioid transformation of macrophages with elongated nuclei, *dashed arrow*, *top*; tapered, spindled macrophages further transformed, *solid arrow*, *left*; and collagen deposition, *arrowhead*, *right*. Magnification at ×200

After the original uptake of mycobacterial organisms by macrophages, a chemokine and cytokine cascade leads to monocyte recruitment, differentiation of macrophages, and epithelioid transformation. The latter is characterized by a large, plumper, more eosinophilic staining cell with somewhat elongated, sole-shaped nuclei (Fig. 1, dashed arrow). In contained tuberculous lesions, especially non-necrotizing ones that do not expand or infiltrate into adjacent bronchial or alveolar airspaces, further change then occurs globally in the appearance of these cells. Cell bodies become less plump and more streaming in shape, with nuclei progressing to a more tapered, spindled appearance (Fig. 1, solid arrow). A similarity between other aspects of nuclear (e.g., chromatin pattern) and cytoplasmic morphology remains between cells in different stages of this continuum. Fibroblasts and associated collagen fiber formation (Fig. 1, arrowhead) are seen in both necrotizing and non-necrotizing lesions, followed by the development of dense bands and sheets of fibrous connective tissue. Eosinophils have been linked to promotion of fibrogenesis [41] but are rarely abundant in lung granulomas in non-human primates (unpublished observations).

### Granulomas exhibit a range of collagen deposition before and after drug treatment

**Fig. 2:**
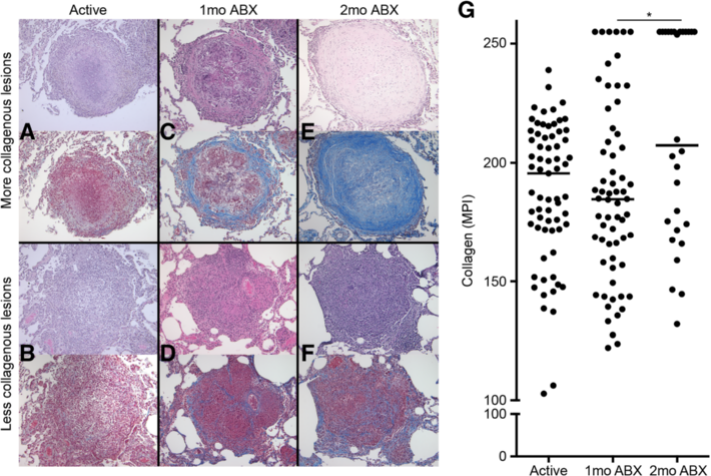
Granulomas exhibit range of collagen deposition before and after drug treatment. Representative sections of histology from granulomas pooled from two TB chemotherapy studies [40, 42], each with H&E staining (*top panel*) and Masson’s trichrome staining (*bottom panel*) for the same lesion. **a** Granuloma from active disease with some peripheral fibrogenesis. **b** Granuloma from active disease with no apparent collagenization. **c** Granuloma from 1-month drug-treated animal with robust peripheral fibrotic cuffing. **d** Granuloma from 1-month drug-treated animal with some central fibrous organization. **e** Fibrotic granuloma from 2-month drug-treated animal. **f** Granuloma from 2-month drug-treated animal with pervasive central fibrous organization. **g** Quantification of all Masson’s trichrome sections. Median pixel intensity was calculated from the blue channel of each micrograph and plotted by stage of drug therapy. *Bars* in each column represent medians. Kruskal–Wallis test: *p* = 0.0285. *p < 0.05

Our laboratory has previously provided histologic evidence for the resolution of TB pathology after 2 months of chemotherapy in cynomolgus macaques [40]. We used Masson’s trichrome staining, a histologic technique for specifically identifying collagen, on pooled granulomas from two previously published drug studies [40, 42] to confirm our hematoxylin and eosin (H&E)-based findings on the same set of animals. We examined if representative lesions have more (Fig. 2a, c, e) or less (Fig. 2b, d, f) collagen present by both histological stains. The more collagenous granuloma from an animal with active disease exhibits a faint ring of peripheral collagen deposition (Fig. 2a), which is not uncommon in tuberculous lesions, while less collagen-rich non-fibrotic granulomas lack this peripheral cuff of collagen (Fig. 2b). After 1 month of drug treatment, some granulomas exhibit a “healing” phenotype [40], as evidenced by a thickening of the peripheral fibrotic cuff and central fibrous organization (Fig. 2c). Other granulomas lack this degree of collagenization but still exhibit some central fibrous organization (Fig. 2d). By 2 months of chemotherapy, there are many lesions whose phenotype is solidly collagenous (Fig. 2e). There are still lesions though that are not completely resolved, and these granulomas are characterized as having some central fibrous development without a robust peripheral cuff (Fig. 2f). These data suggest potentially different cellular mechanisms of tissue repair in tuberculosis, with various degrees of collagenization. We then quantified the photomicrographs for aniline blue-stained collagen to determine how drug treatment modifies collagen deposition (Fig. 2g). We found that granulomas from animals with active disease and 1 month of chemotherapy exhibit a wide range of collagen deposition (Fig. 2g). After 2 months of drug therapy, half of the granulomas are highly collagenous, but the other half displayed lesser degrees of collagenization (Fig. 2g). To put these data within the context of bacterial burden and sterilization, we divided the number of sterile lung granulomas by the total number of lung granulomas to get percentages of sterile lung granulomas for all of the animals in the study (Additional file 1: Figure S1). We found that the frequency of sterile granulomas increases as drug therapy progresses (Additional file 1: Figure S1a) and that this rise is concurrent with a significant increase the presence of collagen-rich granulomas (*p* = 0.0002; Additional file 1: Figure S1b).

### Tuberculous granulomas bear signs of TGFβ-driven fibrosis

**Fig. 3:**
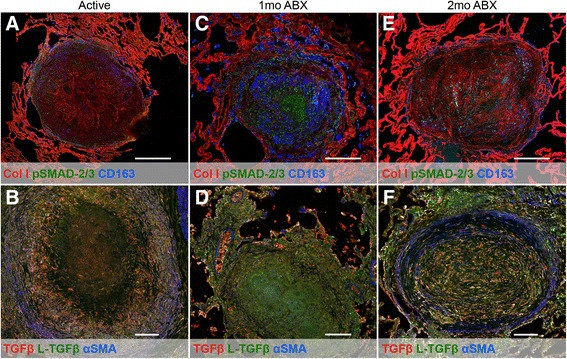
Tuberculous granulomas bear signs of TGFβ-driven fibrosis. *Top panels* feature granulomas stained for collagen I (*red*), phosphorylated SMAD-2/3 (*green*), and CD163 (*blue*). *Bottom panels* feature granulomas stained for TGFβ (*red*), L-TGFβ (*green*), and αSMA (*blue*). Magnification ×200. *Scale bar* represents 500 μm. **a**, **b** Granulomas from animals with active disease. **c**, **d** Granulomas from animals after 1 month of TB chemotherapy. **e**, **f** Granulomas from animals after 2 months of TB chemotherapy

We next wanted to determine whether M2 macrophages and TGFβ were associated with tissue repair. Granulomas from animals with active disease, 1 month of antibiotic (ABX) therapy, and 2 months of ABX were analyzed by multiparameter immunohistochemistry for collagen 1, phosphorylated (p) SMAD-2/3 (an intermediary of TGFβ signaling), and CD163 (a marker of M2-polarized macrophages in primates [5]) or TGFβ, L-TGFβ, and αSMA using appropriate controls (Additional file 2: Figure S2). This approach provides information on the location and quantity of factors upstream of TGFβ induction and TGFβ activation. We found that caseous granulomas from animals with active, untreated disease contained strongly staining fibrils of collagen I that could be visualized in the central region of the lesion while pSMAD-2/3+ and CD163+ cells were present along the periphery of the structure (Fig. 3a). In similar granulomas from untreated animals, active TGFβ was present around the necrotic portion of the lesion (Fig. 3b), an area populated by epithelioid macrophages [5, 43]. Latent TGFβ was observed throughout this granuloma, and αSMA was strongly present in a region of robust peripheral fibrosis. After 1 month of drug treatment, collagen I was detected around the edge of the structure, suggesting mild peripheral fibrotic development (Fig. 3c). Strong pSMAD-2/3 staining was interspersed among the CD163+ cells that were localized towards the center of the structure, a position in which they are not normally found during active disease. Another granuloma from an animal with 1 month of drug therapy exhibited very little αSMA or active TGFβ, except minor staining in the exterior and in an adjacent blood vessel (Fig. 3d). Granulomas from animals 2 months post-chemotherapy displayed plentiful collagen I staining, with both peripheral fibrosis and central fibrotic organization (Fig. 3e). In this particular lesion, pSMAD-2/3 and CD163 appear to co-localize in the fibrosis. We found that αSMA is present in a ring around the periphery of the structure (Fig. 3f). The active and latent forms of TGFβ appear to be intermixed within the lesion. These data overall indicate that active TGFβ is present in these granulomas and drives expression of TGFβ-regulated products including αSMA.

### Active Mtb infection and disease activates TGFβ and suppresses SMAD-2/3 signaling

**Fig. 4:**
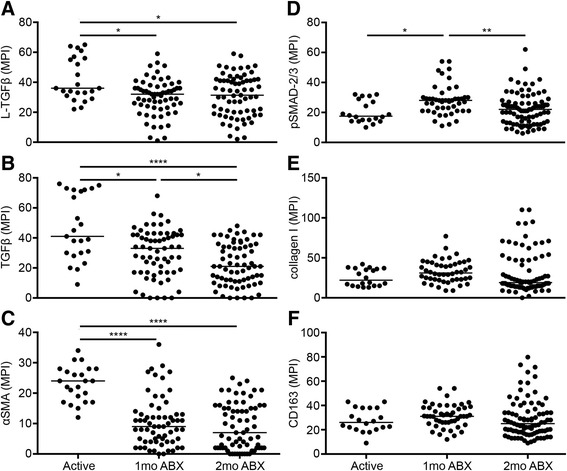
Active Mtb infection and disease activates TGFβ and suppresses SMAD-2/3 signaling. **a**–**f** Quantification of IHC. Median pixel intensity (MPI) was calculated from *red*, *green*, and *blue color* channels of each micrograph and plotted by stage of drug therapy. *Bars* in each column represent medians. Kruskal–Wallis test had *p* values of less than 0.0001 for TGFβ and αSMA, pSMAD-2/3 (0.0006), L-TGFβ (0.0148), and collagen I and CD163 (not significant). *p < 0.05. **p < 0.01. ***p < 0.001. ****p < 0.0001

We quantified median pixel intensity (MPI) from a collection of granulomas to elucidate how latent and active TGFβ, αSMA, pSMAD2/3, collagen, and CD163 expression change over the three time points we are examining. We found an initial decrease in latent TGFβ levels at the initiation of drug treatment, but unchanged levels between 1 and 2 months of drug therapy (Fig. 4a). In contrast, active TGFβ appeared to decline in animals over the full 2-month course of drug therapy (Fig. 4b), while the trend in αSMA expression was consistent with trend observed for latent TGFβ (Fig. 4c). Interestingly, levels of phosphorylated SMAD-2/3 were higher after 1 month of chemotherapy but then decreased by 2 months of antibiotics (Fig. 4d). Relative amounts of collagen I and CD163 do not change between following antibiotic treatment (Fig. 4e–f). These data further demonstrate the role that active TGFβ is playing in drug-promoted tissue repair.

**Fig. 5:**
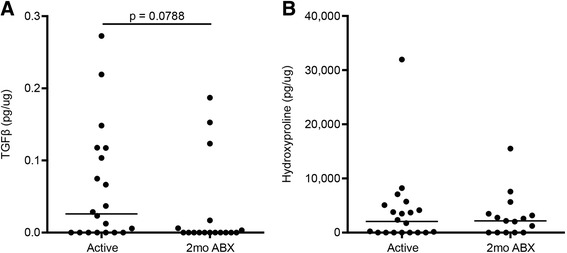
Biochemical evidence of TGFβ and collagen. **a** ELISA for active TGFβ. TGFβ in granulomas was converted into pg and then normalized by the total amount of protein in each granuloma as determined by BCA assay. Wilcoxon signed-rank test was used to compare granulomas from active disease to granulomas from 2 months of drug treatment. **b** Hydroxyproline assay. Collagen present in granulomas was determined using the hydroxyproline assay. Values were converted into pg and then normalized by the total amount of protein in each granuloma as determined by BCA assay

We used immunologic and biochemical assays to confirm our immunohistochemical analysis of TGFβ and collagen. Consistent with our immunohistochemical data, enzyme-linked immunosorbent assays (ELISAs) for active TGFβ demonstrated greater quantities of TGFβ in granulomas from animals during active disease and lesser in animals after 2 months of drug treatment (Fig. 5a). Collagen levels were measured by hydroxyproline assay (Fig. 5b) and were unchanged between treated and untreated animals, coinciding with our microscopic data (Fig. 4f).

### TGFβ is strongly associated with collagen I expression

**Fig. 6:**
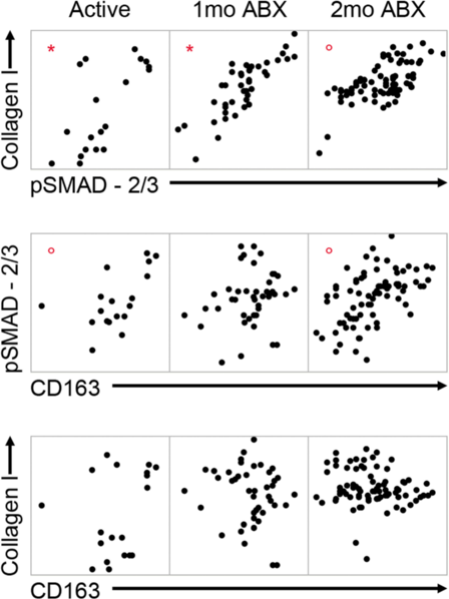
TGFβ is strongly associated with the presence of collagen I. IHC values for each granuloma were plotted against one another to assess association between collagen I, TGFβ signaling, and M2 macrophages. Values were transformed, and Pearson’s test for parametric correlation was used to determine significance. Significant correlations are denoted by *red symbols. Open circles* are significant but *r* < 0.7, while *asterisks* indicate that *r* > 0.7

We used our quantitative immunohistochemical data (Fig. 4) to perform pairwise correlations to identify the relationships between these factors. At all study time points, collagen I and pSMAD-2/3 exhibit a robust positive association (Fig. 6), especially during active disease and 1 month of drug therapy. In these time points, we found that over 50 % of the variability in collagen I levels was explained by pSMAD-2/3 (Pearson’s *r* > 0.7). In granulomas from animals with active disease, or 2 months of drug treatment, there is a weak but significant association between pSMAD-2/3 and CD163 (Fig. 6). However, CD163 and collagen I are not statistically associated at any time point of the study (Fig. 6). This is in contrast to active TGFβ, latent TGFβ, and αSMA, which are all strongly and positively associated with one another at all time points (Additional file 3: Figure S3).

## Discussion

The goal of this study was to identify molecular and cellular markers associated with fibrotic resolution in experimental tuberculosis infection. Tuberculosis produces a wide range of pulmonary pathologies through infection and disease, which typically result in chronic fibrocaseous disease [6, 7]. Drug treatment reduces bacterial burden and allows for tissue repair, although this does not quickly alter the pathology remaining in the lung as demonstrated by computed tomography in both human and non-human primates [44]. Chemotherapy additionally leaves individuals vulnerable to relapse; more than half of smear positive TB cases have been previously treated for TB [45]. Relapse was originally thought to be cause by endogenous sources of Mtb remaining after drug treatment, but 75 % of relapse cases in a high TB burden cohort were exogenously reinfected [46]. Why is this population prone to reinfection after successful chemotherapy? The risk of developing active TB is about 4.5 times higher in patients with chronic interstitial lung disease and idiopathic pulmonary fibrosis [47, 48]. This could be due to the specific pathology of fibrotic lung tissue as it may promote colonization and establishment of infection [49]. The residual scar tissue left after drug therapy and loss of normal lung architecture could play roles in increasing the risk of relapse. Therefore, understanding the mechanism of fibrogenesis in TB and ameliorating tissue damage after drug treatment could reduce the risk of reinfection and help shrink the pool of infected individuals.

Several cell types are likely to play significant roles in driving fibrotic processes in granulomas, but it has been difficult to identify the specific cells that are the most important drivers of fibrosis. Histologic assessment can be used to evaluate the cellular responses to infection from a morphological perspective and can provide insights into lesion development and resolution. Identifying the cellular origin of some spindle-type cells in granulomas is not always possible, but histologic examination can provide important inferences as to whether these cells arose from epithelioid macrophages or collagen-producing fibroblasts. Based on our histologic observations and immunohistochemical analyses, we hypothesize that collagen-producing fibroblast-like cells may originate from several sources, and in some cases not from fibroblasts recruited from nearby tissues, but from non-fibroblast-like cells that have undergone a process similar to endothelial–mesenchymal progression. Additionally, circulating monocyte-derived cells (fibrocytes) can have phenotypes reminiscent of the phenotypes we have identified [50, 51]. Given the ability of some circulating monocytes to differentiate into fibroblast-like cells instead of macrophages, the question of whether subsequent differentiation between macrophages and fibroblasts can occur should be considered. The wide range in collagen presence and the appearance of various collagenous phenotypes suggest that different mechanisms for fibrotic resolution occur in tuberculous granulomas. Some granulomas manifest both peripheral and central fibrous organization, while others demonstrate only central collagenization, or none at all. Future studies should seek to correlate these divergent repair mechanisms to lesion-specific Mtb burden to determine efficacy of clearance and elucidate the immunologic processes of each.

**Fig. 7:**
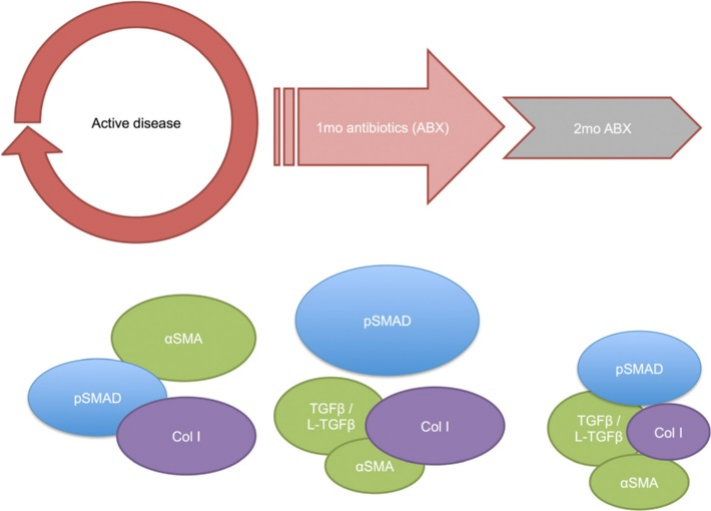
Model that demonstrates the TGFβ-associated processes explores in this study. The *red arrow* around active disease represents processes that activate fibrotic cytokines, which then diminish during the early course of drug therapy (*smaller arrows* that are *less red*). αSMA and TGFβ drop, represented in *green*, while pSMAD-2/3 appears to rise and fall, in *blue*. Levels of collagen I do not change, shown in *purple*

Our observations that latent TGFβ changes only slightly between granulomas from animals with active disease and drug-treated animals suggest that a pool of latent TGFβ remains in the lung even after Mtb is cleared. Latent TGFβ is maintained in the lung epithelium by latent TGFβ-binding proteins where it can remain until activated [52]. Our data indicate that conditions within granulomas activate TGFβ, an anti-inflammatory cytokine associated with fibrosis (Fig. 7). This molecule exerts biological action in the granuloma, as is supported by the presence of αSMA in direct proportion to TGFβ. The lower levels of TGFβ and αSMA in granulomas of animals on drug therapy suggest that therapy itself does not activate more TGFβ and that the amount activated during active TB is utilized for tissue repair. Interestingly, phosphorylated SMAD-2/3 is lower during active disease, even though active TGFβ levels are high. Since SMAD-2/3 is phosphorylated when cells come into contact with active TGFβ [20], this suggests that live Mtb somehow blocks SMAD-2/3 activation even in the presence of active TGFβ. The presence of αSMA, also an indicator of the cellular effects of TGFβ, could be explained by the utilization of alternative TGFβ-stimulated pathways [53, 54]. The alternative TGFβ signaling pathways may be prominent in disease, and since the SMAD-2/3 pathway only becomes used in tissue repair once Mtb is cleared, the SMAD-2/3 pathway may be preferential for healing and clearance and the alternative pathways preferential for TB pathogenesis. Collagen I levels are similar between granulomas with active disease and drug treatment, suggesting that treatment only promotes and does not exacerbate fibrosis. Collagen I is strongly associated with active TGFβ; over 50 % of the variability in levels of collagen I is explained by TGFβ signaling through SMAD-2/3, indicating that active TGFβ is likely the main molecular driver of fibrosis and tissue repair in tuberculous lesions. CD163 and collagen I are not associated in this model, implying that the CD163+ macrophages are not major producers of collagen I. Macrophages in granulomas are not a uniform population of cells [5], and other CD163− macrophage subsets could be responsible for collagen production in the granuloma. Unfortunately, there are no pan-macrophage markers for primates, and our efforts to identify the specific population of cells responsible for collagen expression are ongoing [5]. Collagen appears to stream from the epithelioid macrophages into the caseous necrotic center. Interestingly, this population of cells is not comprised of the M2-polarized CD163+ macrophages we expected would correlate with fibrosis, but instead appear to be M1-polarized cells [5], suggesting the capacity of these cells may be more diverse than previously known. Active TGFβ was mostly localized in the epithelioid macrophage region of the granulomas as well. Alternatively, macrophage-expressed products could support collagen production by neighboring fibroblasts. These cells could potentially be contributors to collagenization, as well as surrounding lung epithelial cells transformed by active TGFβ. Further work will include markers for these types of cells and determine their association with collagen production in the granuloma.

## Conclusions

Fibrosis occurs in TB lesions during and after drug treatment. The goal of this project was to determine the cell types and molecules associated with fibrosis in non-human primates with TB. We provide additional evidence of activated TGFβ being present in lesions from *M. tuberculosis*-infected lung tissue. Collagen production in active disease and TB chemotherapy is strongly associated with TGFβ, suggesting its role as the chief cytokine driving tissue repair. Future studies will seek to further explore these results mechanistically with hopes of developing adjunct treatment to minimize, or possibly reverse, scar formation after TB chemotherapy.

## Methods

### Animals

The Institutional Animal Care and Use Committee of University of Pittsburgh approved all experiments. The animals were housed and maintained in accordance with standards established in the Animal Welfare Act and the Guide for the Care and Use of Laboratory Animals.

#### Infections

Cynomolgus macaques (4–9 years of age) imported from China (Valley Biosystems, Sacramento, CA) were used for these studies (*n* = 21). Monkeys were infected via bronchoscope with 25 CFU of *M. tuberculosis* Erdman strain. Using published criteria, monkeys were determined to have active or latent TB by 6–8 months post-infection and were randomized to treatment or non-treatment groups; treatment was initiated when monkeys developed active TB, as determined by clinical and microbiologic signs [43, 55].

#### Drug therapy

The samples described in the current study were obtained from macaques in a previously published study from our laboratory [40, 42]. Monkeys with active disease were as follows: untreated (*n* = 9, 110 granulomas), treated with isoniazid (INH) and rifampin (RIF) for 1 month (*n* = 2, 22 granulomas) or 2 months (*n* = 5, 91 granulomas), or treated with INH, RIF, and metronidazole (MTZ) for 1 month (*n* = 3, 154 granulomas) or 2 months (*n* = 5, 93 granulomas).

Necropsies were performed as previously described [35, 51]. Individual granuloma and lung samples were taken from each monkey from sites of infection and surrounding tissue. Portions of these samples were homogenized into single cell suspension before storing at −80 °C for ELISA and hydroxyproline assays, while another portion was formalin-fixed paraffin-embedded for histology.

### H&E staining

Formalin-fixed paraffin-embedded tissue sections were cut and stained with Harris hematoxylin modified (Sigma-Aldrich, St. Louis, MO) and eosin Y solution (Sigma-Aldrich, St. Louis, MO). Slides were deparaffinized in deionized water. Slides were then stained with hematoxylin for 3 min. Slides were rinsed under running tap water, rinsed with 70 % ethanol, and then stained with eosin for 3 min. Slides were rinsed and dehydrated in ethanol, cleared in xylene, and then mounted. Criteria for characterizing granulomas were based on size and shape, type of granuloma, and cellular composition. A veterinary pathologist who is an expert in macaque tuberculosis (ECK) performed all histologic analyses.

### Masson’s trichrome

Formalin-fixed paraffin-embedded tissue sections were stained with Masson’s trichrome to identify connective tissue, muscle, and collagen fibers (Sigma-Aldrich, St. Louis, MO). Slides were deparaffinized to deionized water. Slides were then immersed in Bouin’s solution overnight at room temperature to intensify the subsequent staining. Slides were washed with tap water then stained with Harris hematoxylin solution (Sigma-Aldrich, St. Louis, MO) for 5 min. Slides were washed again in running tap water for 5 min, rinsed in deionized water, and stained in Biebrich scarlet-acid fuchsin for 5 min. Slides were rinsed in deionized water and placed in phosphotungstic and phosphomolybdic acid solution for 5 min. Slides were moved to Aniline Blue solution for 5 min and then placed in 1 % acetic acid solution for 2 min. Slides were rinsed in deionized water, dehydrated through alcohol, cleared in xylene, and then mounted.

### IHC

Tissue sections were stained for collagen I (rabbit polyclonal, Abcam, Cambridge, MA, 1:50 dilution), pSMAD-2/3 (goat polyclonal, Santa Cruz Biotechnology, Dallas, TX, 1:10 dilution), CD163 (mouse clone 10D6, Neomarkers, Fremont, CA, 1:30 dilution), L-TGFβ (goat polyclonal, R&D Systems, Minneapolis, MN, 1:10 dilution), TGFβ (chicken polyclonal, R&D Systems, Minneapolis, MN, 1:10 dilution), and αSMA (mouse clone 1A4, Thermo Fisher, Pittsburgh, PA, 1:100 dilution). Antigen retrieval and staining were done as previously described [5]. Briefly, formalin-fixed paraffin-embedded tissues samples were deparaffinized in xylene and rehydrated in ethanol. Samples were then placed into a pressure cooker with boiling antigen retrieval buffer (Tris–HCl, EDTA, Tween-20) for 7 min. After allowing for the slides to cool, the sections were blocked with 2 % fetal bovine serum in phosphate-buffered saline for 30 min. Antibodies and fluorescent tags were incubated on each sample for 1 h with washes in between with phosphate-buffered saline with 0.2 % Tween-20. Prolong Gold Mounting Medium with DAPI (Invitrogen) was then applied to the slides, which were then cured in the dark overnight before being imaged.

### Quantification of histology

Trichrome- and H&E-stained sections were imaged using Provis fluorescent microscope (Olympus America, Center Valley, PA) and fluorescently stained slides visualized with a FluoView 1000 confocal microscopes (Olympus). For images used for quantitative imaging, care was taken to keep the camera settings constant between granulomas and animals. These images were then saved as 24-bit TIFF files and read into the language R via the package “EBImage” from Bioconductor (http://www.bioconductor.org/packages/release/bioc/html/EBImage.html). For the trichrome slides, the blue channel only was isolated. Red, green, and blue channels were pulled from the immunohistochemistry (IHC) slides. The median pixel intensity for each channel was then saved and exported to Microsoft Excel (Microsoft, Redmond, WA).

### ELISAs

Active TGFβ-1 in granuloma homogenates was measured by using a commercial ELISA (eBioscience, San Diego, CA) according to the manufacturer’s instructions. Briefly, a high-affinity protein-binding plate was coated with a capture antibody overnight and blocked with assay diluent for an hour before adding standards and undiluted granuloma homogenates for overnight incubation. TGFβ was detected the next day using a biotinylated detection antibody and streptavidin-HRP, and the absorbance was immediately measured at 450 nm. Total protein of the same samples was quantified by Pierce BCA Protein Assay (Thermo Scientific, Pittsburgh, PA), where granuloma homogenates were added to BCA Working Reagent, and the absorbance measured at 562 nm after 30 min at 37 °C. Levels of TGFβ were normalized to total protein in granuloma homogenates.

### Hydroxyproline assay

Collagen was detected in homogenized granulomas by using a commercial hydroxyproline kit (Sigma-Aldrich, St. Louis, MO). Briefly, samples were mixed with hydrochloric acid and hydrolyzed at 120 °C for 3 h. These samples and standards were then transferred to a 96-well plate and dried. Chloramine T/oxidation buffer was added to wells and incubated at room temperature for 5 min. To this, diluted DMAB reagent was added and incubated at 60 °C for 90 min. Absorbance at 560 nm was then measured. Levels of collagen were normalized to total protein in granuloma homogenates.

### Statistics

Quantitative data from Masson’s trichrome staining and immunohistochemical staining were visualized using Prism (Graphpad, La Jolla, CA). Analysis of these data was done using the tests indicated, typically a one-way ANOVA and a multiple comparison test for either parametric or non-parametric data. *p* values were significant if less than 0.05. Further analysis of the immunohistochemical staining was performed with JMP (SAS, Cary, NC). Correlations between markers in histological stains were performed using the multivariate function, and scatter plots were generated. Significance was determined using pairwise correlations with the strength of the relationship given as Pearson’s *r*.

## Additional files

Additional file 1: Figure S1. Antibacterial chemotherapy promotes collagen-associated bacterial clearance. **a** The number of lung granulomas with viable bacterial growth were divided by the total number of lung granulomas and plotted for each animal in the three groups. Each dot represents one animal. Bars represent medians. **b** The median of the median pixel intensity of the aniline blue staining was determined. This was then plotted against the percent of lung granulomas with viable bacteria. Significance of the interaction between percent of Mtb + lung granulomas and collagen staining (p) and strength of interaction (R^2^) are shown. Each dot represents one animal, and colors match the colors of the treatment groups in part A of this figure. Dotted lines represent 95 % confidence intervals. (TIF 271 kb) Additional file 2: Figure S2. Negative and positive controls for immunohistochemistry. Left panels feature negative controls for IHC and right panels feature blood vessels, which should serve as bright signals for most of the markers. Negative controls did not receive primary antibodies, but received secondary antibodies and were imaged using the same settings as the slides for the study. **a-b** Negative control and blood vessel for the first panel. **c-d** Negative control and blood vessel using the second panel. (TIF 7053 kb) Additional file 3: Figure S3. Active TGFβ is highly associated with its latent form and αSMA. IHC values for each granuloma were plotted against one another to assess association between TGFβ, L-TGFβ, and αSMA—which is produced by cells activated by TGFβ. Values were transformed, and Pearson’s test for parametric correlation was used to determine significance. Significant correlations are denoted by red symbols. Open circles are significant but r < 0.7, while asterisks indicate that r > 0.7. (TIF 843 kb)

## Abbreviations

ABX: antibiotics
H&E: hematoxylin and eosin
IHC: immunohistochemistry
INH: isoniazid
Mtb: *Mycobacterium tuberculosis*
MTZ: metronidazole
RIF: rifampicin
TB: tuberculosis
TGFβ: transforming growth factor-β
αSMA: α-smooth muscle actin
