# Supplementary figures and images for: Active transforming growth factor-β is associated with phenotypic changes in granulomas after drug treatment in pulmonary tuberculosis
Source: Fibrogenesis Tissue Repair. 2016 Apr 27;9:6. doi: 10.1186/s13069-016-0043-3 (PMC4855369; doi:10.1186/s13069-016-0043-3)

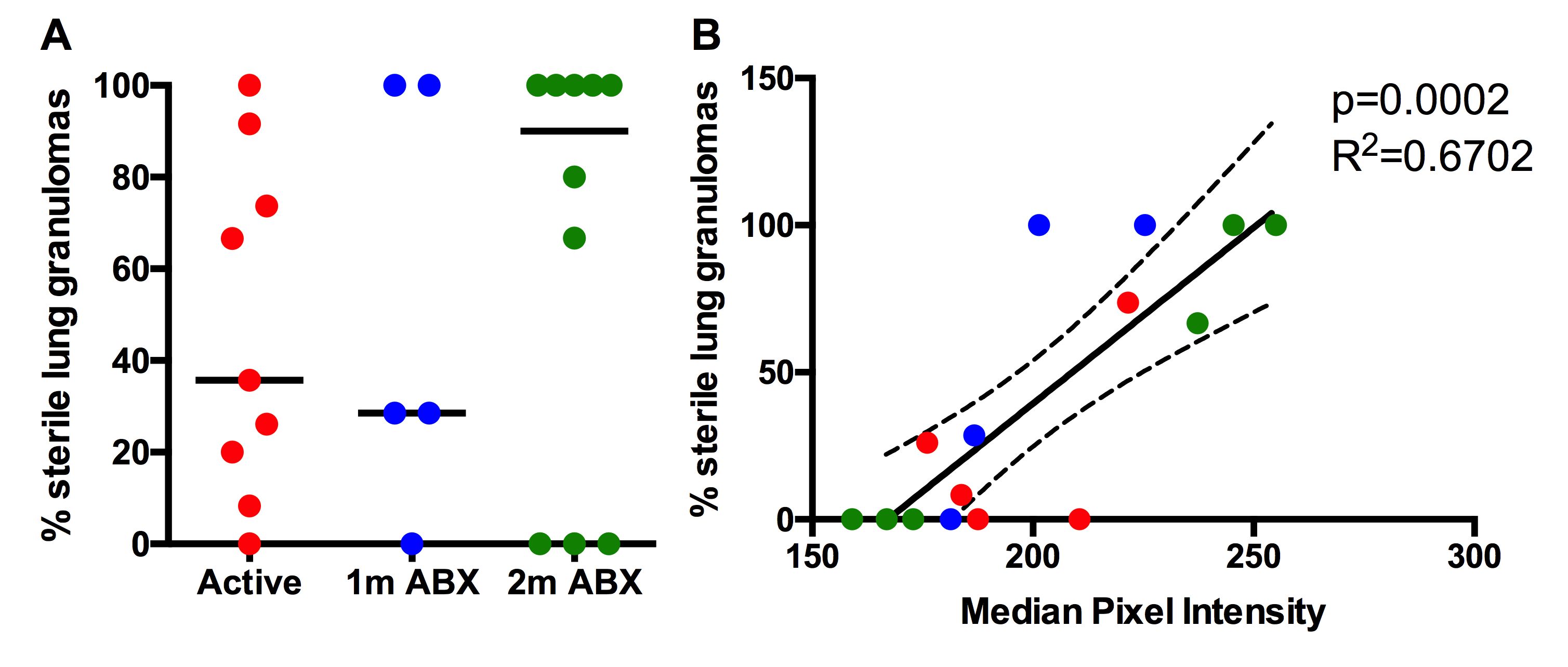

Supplement: Additional file 1: Figure S1. — Antibacterial chemotherapy promotes collagen-associated bacterial clearance. a The number of lung granulomas with viable bacterial growth were divided by the total number of lung granulomas and plotted for each animal in the three groups. Each dot represents one animal. Bars represent medians. b The median of the median pixel intensity of the aniline blue staining was determined. This was then plotted against the percent of lung granulomas with viable bacteria. Significance of the interaction between percent of Mtb + lung granulomas and collagen staining (p) and strength of interaction (R2) are shown. Each dot represents one animal, and colors match the colors of the treatment groups in part A of this figure. Dotted lines represent 95 % confidence intervals. (TIF 271 kb) [file 13069_2016_43_MOESM1_ESM.tif]

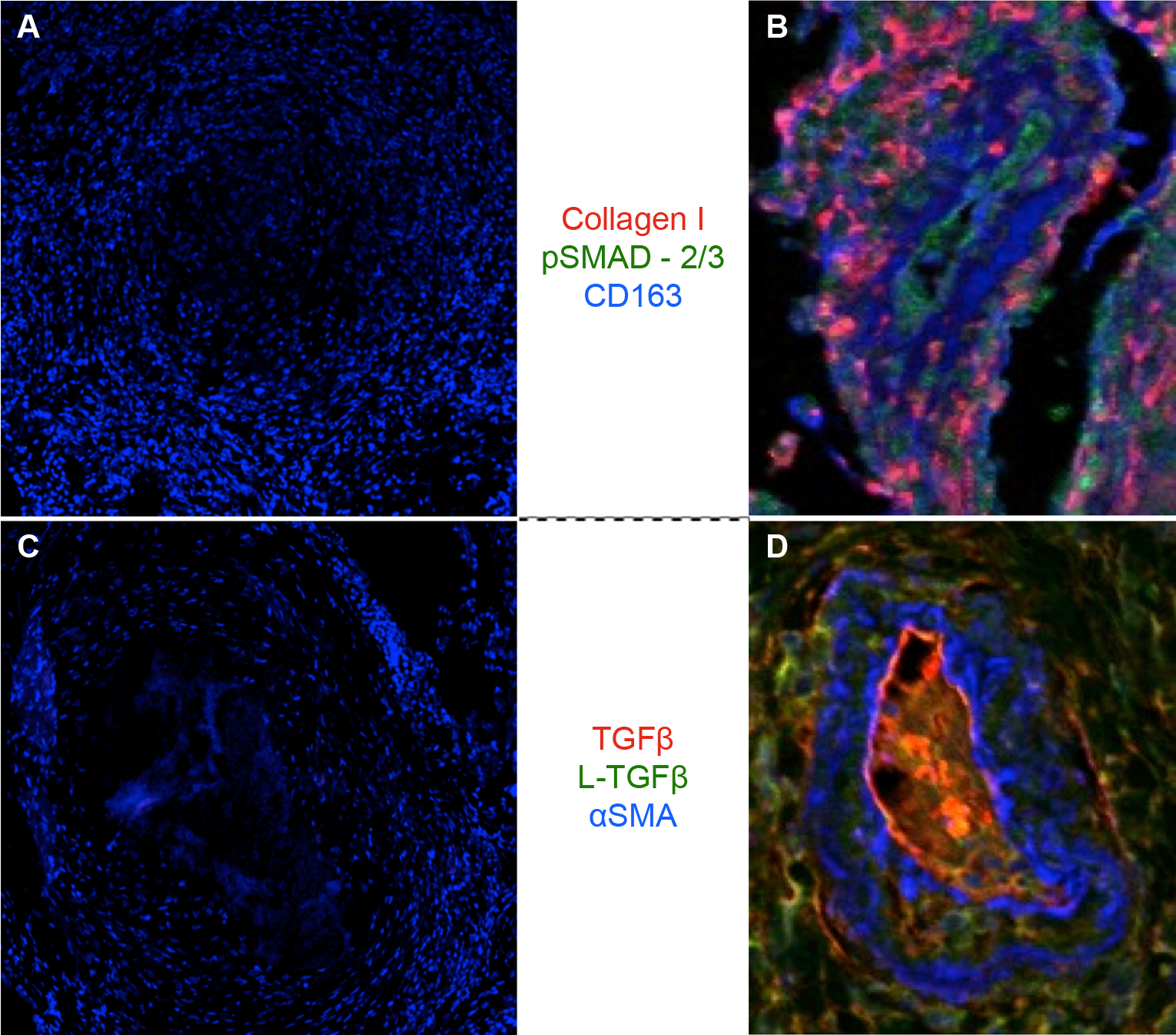

Supplement: Additional file 2: Figure S2. — Negative and positive controls for immunohistochemistry. Left panels feature negative controls for IHC and right panels feature blood vessels, which should serve as bright signals for most of the markers. Negative controls did not receive primary antibodies, but received secondary antibodies and were imaged using the same settings as the slides for the study. a-b Negative control and blood vessel for the first panel. c-d Negative control and blood vessel using the second panel. (TIF 7053 kb) [file 13069_2016_43_MOESM2_ESM.tif]

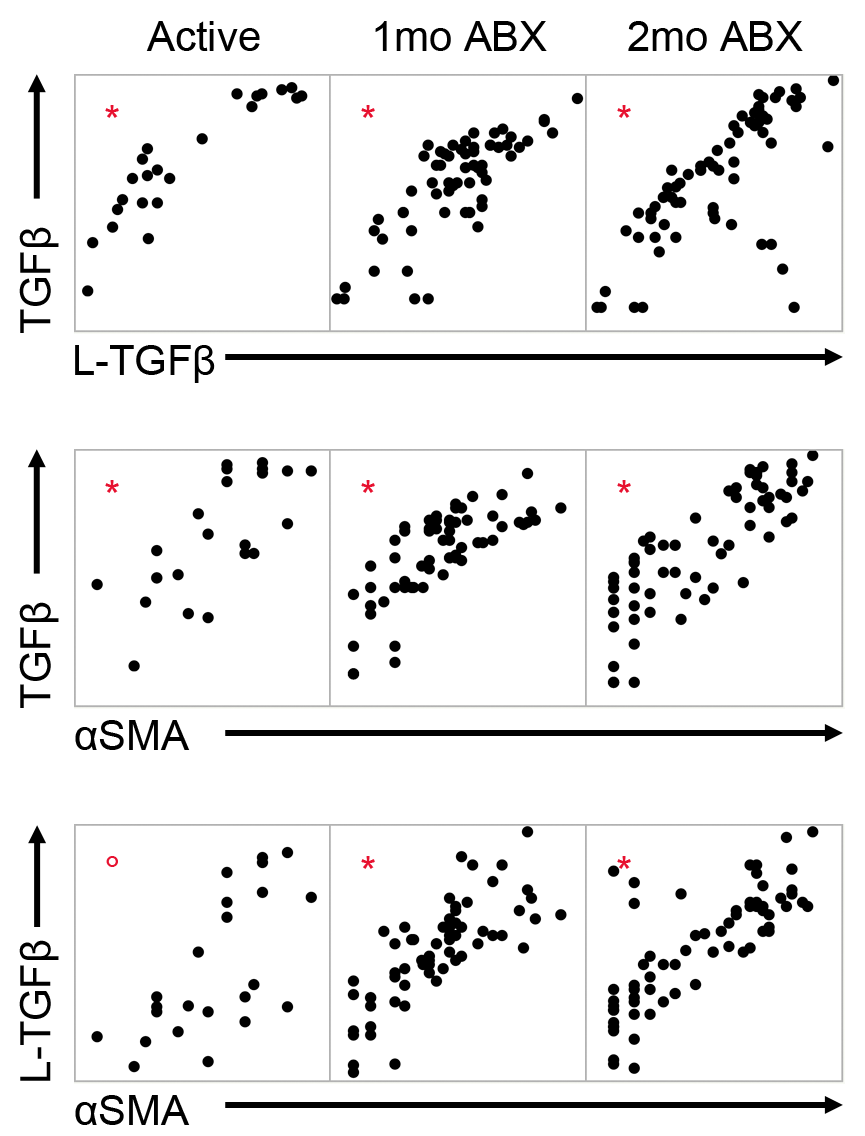

Supplement: Additional file 3: Figure S3. — Active TGFβ is highly associated with its latent form and αSMA. IHC values for each granuloma were plotted against one another to assess association between TGFβ, L-TGFβ, and αSMA—which is produced by cells activated by TGFβ. Values were transformed, and Pearson’s test for parametric correlation was used to determine significance. Significant correlations are denoted by red symbols. Open circles are significant but r < 0.7, while asterisks indicate that r > 0.7. (TIF 843 kb) [file 13069_2016_43_MOESM3_ESM.tif]
